# Determination of Multiple Active Components in Mume Fructus by UPLC-MS/MS

**DOI:** 10.3390/metabo15050312

**Published:** 2025-05-06

**Authors:** Nannan Li, Jingyi Yue, Rui Wang

**Affiliations:** 1 Wuhu Institute of Technology, Wuhu 241009, China; linn@whit.edu.cn (N.L.); yuejy@whit.edu.cn (J.Y.); 2 Life and Health Engineering Research Center of Wuhu, Wuhu 241009, China; 3 Dali University, Dali 671000, China

**Keywords:** Mume Fructus, PCA, TCM, UPLC-QTRAP-MS/MS

## Abstract

**Background:** This study presents a sensitive method for the simultaneous determination of organic acids, flavonoids, and amino acids in Mume Fructus (MF) using ultra-performance liquid chromatography coupled with triple-quadrupole linear ion-trap tandem mass spectrometry (UPLC-QTRAP-MS/MS). **Methods:** Analysis was performed on a UPLC system (Shimadzu, Kyoto, Japan) equipped with a quaternary pump solvent management system, an online degasser, a triple-quadrupole mass detector, and an autosampler. An Agilent ZORBAX SB-C18 column (3.0 mm × 100 mm, 1.8 µm) was used for chromatographic analyses. The mobile phase was distributed between 0.2% aqueous formic acid (A) and 0.2% formic acid acetonitrile (B) at a velocity of 0.2 mL/min. The gradient evolution protocol was 0–2 min at 90–70% B; 3–7 min at 70–50% B; 7–10 min at 50–20% B; 10–14.5 min at 20–90% B; and 14.5–17 min at 10% B. **Results:** The method was validated for matrix effects, linearity, limits of detection/quantification, precision, repeatability, stability, and recovery of target components. It effectively determined all target compounds in 12 MF batches from different drying methods. **Conclusions:** Principal component analysis (PCA) of 47 active components was conducted to evaluate MF quality comprehensively. The proposed method serves as a reliable approach for assessing the consistency of MF’s quality and therapeutic efficacy.

## 1. Introduction

Mume Fructus (MF) is the dried immature fruit of *Prunus mume* (Sieb.) Sieb. et Zucc, and its whole herb was listed in the Chinese Pharmacopoeia [1]. It is commonly called a sour plum in China and grown in temperate areas like Yunnan, Fujian, and Sichuan [2,3]. In traditional Chinese medicine (TCM), MF has been widely used to treat gastrointestinal disease like ulcerative colitis [4,5]. Nowadays, more and more studies have shown that the active compounds of MF have various pharmacological actions, including anti-oxidative [6], anti-cancer [7], analgesic [8], anti-inflammatory [9], neuroprotective [10] and antibacterial actions [11]. The hundreds of chemical components of MF result in its biological activities. MF contains different kinds of compounds, including organic acids, sugars, flavonoids, terpenes, amino groups, nucleosides, and other active chemical components [12].

Because of its significant medical value, MF and its relevant products are used in large amounts. According to the literature, the production of MF is nearly hundreds of thousands of tons every year. So, it is necessary to develop a rapid method for the simultaneous determination of multiple compounds as well as for the quality control of MF. Through a continuous research program addressing the isolation, structural characterization, and pharmacological evaluation of natural products, over 100 chemical compositions have been isolated and identified from the fruits and flower heads of the plants of MF [13,14,15]. At present, the organic acid, flavonoid, and amino acid composition is used as the index to determine the quality of medicinal ingredients, such as citric acid, rutin, and aspartic acid [16,17,18]. Many methodologies have been reported to control the quality of MF. For instance, high-performance liquid chromatography with an ultraviolet detector (HPLC-UV) has been used for the determination of organic acid [19], and HPLC with an evaporative light scattering detector (HPLC-ELSD) was employed to quantitate saponins and iridoid glucosides [20]. However, there are several shortcomings such as the relatively low sensitivity of ELSD and the inaccuracy of chromatographic peaks only determined by retention time. With the development of analytical technology, HPLC-DAD–electrospray ionization mass spectrometry (ESI-MS) was proposed to analyze multiple types of bioactive constituents. Ultrafast LC (UFLC) combined with quadrupole/linear ion trap (QTRAP) and tandem MS (MS/MS) uses a multiple-reaction monitoring (MRM) mode to scan the chemical constituents of the fragments, providing high sensitivity and selectivity and allowing for analyses to be performed rapidly [21].

Recently, we successfully identified chemical components from MF using UPLC-Q-TOF technology [22]. Therefore, a reliable, sensitive and selective LC-MS/MS method was successfully established for the simultaneous quantification of 47 active components (10 organic acids, 4 flavonoids, 1 cyanoside, 1 anthocyanin, 1 carbohydrate, 20 amino acids, and 10 nucleosides) in one run cycle. Moreover, from the results of cluster analysis, it can be concluded that the different processing methods have a significant impact on the chemical compositions in MF. The proposed approach could be readily utilized as a comprehensive approach for determining the consistency of the quality and therapeutic efficacy of MF.

## 2. Materials and Methods

### 2.1. Chemicals, Reagents, and Materials

Forty-seven chemical standards were used, including the following: benzoic acid (1), quercitrin (2), caffeic acid (3), fumaric acid (4), chlorogenic acid (5), protocatechuic acid (6), succinic acid (7), quininic acid (8), rutin (9), citric acid (10), malic acid (11), kaempferol (12), ursolic acid (13), amygdalin (14), cyanidin-3-O-glucoside chloride (15), apigenin (16), hydroxymethylfurfural (17), *L*-alanine (18), *L*-serine (19), *L*-aspartic acid (20), *L*-asparagine (21), *L*-valine (22), *L*-glutamic acid (23), *L*-isoleucine (24), *L*-methionine (25), *L*-arginine (26), *L*-histidine (27), *L*-threonine (28), *L*-phenylalanine (29), *L*-leucine (30), *L*-cystine (31), *L*-hydroxyproline (32), *L*-tyrosine (33), *L*-tryptophan (34), *L*-proline (35), *L*-lysine hydrochloride (36), γ-aminobutyric acid (37), guanine (38), adenine (39), uracil (40), hypoxanthine (41), thymidine (42), guanosine (43), inosine (44), uridine (45), adenosine (46), and cytidine (47). The purity of all standard components was ≥ 98%. *L*-alanine, *L*-serine, *L*-aspartic acid, *L*-asparagine, *L*-valine, *L*-glutamic acid, *L*-isoleucine, *L*-methionine, *L*-arginine, *L*-histidine, *L*-threonine, *L*-phenylalanine, *L*-leucine, *L*-cystine, *L*-hydroxyproline, *L*-tyrosine, *L*-tryptophan, *L*-proline and *L*-lysine hydrochloride were purchased from National Institutes for Foods and Drugs (Beijing, China). The remainder were obtained from Shanghai Yuanye Biotechnology (Shanghai, China). Chromatography-grade methanol and acetonitrile were purchased from Merck (Darmstadt, Germany). Ultrapure water was obtained using a Milli-Q™ purification system (Millipore, Billerica, MA, USA).

Samples were collected in 2021, and samples S1–S12 samples were smoked with pine wood, at a temperature of 60–80° for 72 h. S13–S18 were dried in an oven at a low temperature of 60 °C for 30 h. Sample information can be found in Table 1.

### 2.2. Preparation of Standard Solutions

Forty-seven standard substances were prepared by dissolution in ultrapure water, and their concentrations (in mg/mL) were as follows (numbers in parentheses correspond to chemical number as listed in Section 2.1: (1) 0.12, (2) 0.11, (3) 1.21, (4) 2.64, (5) 1.30, (6) 1.31, (7) 2.40, (8) 2.69, (9) 1.41, (10) 12.31, (11) 6.12, (12) 0.41, (13) 2.01, (14) 0.50, (15) 1.21, (16) 2.58, (17) 0.98, (18) 0.99, (19) 1.26, (20) 2.80, (21) 1.56, (22) 1.08, (23) 0.80, (24) 0.28, (25) 0.11, (26) 0.20, (27) 0.95, (28) 0.61, (29) 0.15, (30) 0.35, (31) 0.55, (32) 0.40, (33) 1.05, (34) 0.91, (35) 0.36, (36) 0.16, (37) 0.66, (38) 0.25, (39) 0.37, (40) 0.18, (41) 0.87, (42) 0.46, (43) 0.21, (44) 0.37, (45) 0.68, (46) 0.19, and (47) 0.13. A mixed standard stock solution containing all 47 standard substances was serially diluted with ultrapure water to the required concentration for the establishment of calibration curves. All solutions were stored at 4 °C and then passed through a 0.22 μm membrane.

### 2.3. Preparation of Sample Solutions

A total of 1.0 g of sample powder (powder, sieve 3) was taken, and 30 mL of ultrapure water was added, followed by weighing the mass and sonication for 30 min at room temperature. The same solution was used to replace the extraction system after solar event loss data changed to volatile. The supernatant was removed, centrifuged (13,000 r/min) for 10 min, filtered through 0.22 μm filter membrane, and then the continued filtrate was taken [22]. The extraction method we used aimed to maximize the extraction of active components from Mume Fructus while minimizing potential losses and interferences. The ultrasound extraction method ensures that the samples are fully dissolved and effectively releases the active components, simplifying the subsequent analytical steps.

### 2.4. UPLC–MS/MS Instrumentation and Conditions

Analysis was performed on a UPLC system (Shimadzu, Kyoto, Japan) equipped with a quaternary pump solvent management system, an online degasser, a triple-quadrupole mass detector, and an autosampler. An Agilent ZORBAX SB-C18 column (3.0 mm × 100 mm, 1.8 µm) was used for chromatographic analyses. The mobile phase was distributed between 0.2% aqueous formic acid (A) and 0.2% formic acid acetonitrile (B) at a velocity of 0.2 mL/min. The gradient evolution protocol was 0–2 min at 90–70% B; 3–7 min at 70–50% B; 7–10 min at 50–20% B; 10–14.5 min at 20–90% B; and 14.5–17 min at 10% B. The optimized parameters for MS for the 47 target components are shown in Table 2. All MS data were analyzed using Analyst 1.6.2 (AB SCIEX).

### 2.5. Multivariate Statistical Analyses

PCA was used to visualize the similarity or differences in multivariate data. PCA represents an unsupervised pattern recognition technology that can be used to transfer multiple variables through linear transformations to select a few important variables. To observe the classifications of experimental samples, PLS-DA, supervised by Simca-p 14.1, was conducted to perform PCA using data from 47 analytes, to discover the different chemical compositions of each sample.

## 3. Results

### 3.1. MS Condition Optimization

In the UPLC-MS experiment, we identified the compounds using databases such as the Human Metabolome Database (HMDB) and PubChem, in conjunction with mass spectrometry fragmentation patterns and retention times. Additionally, we used Progenesis QI software (2.1.2) for data analysis to assist in confirming and identifying the compound names. Separate solutions (about 100 ng/mL) of all standard compounds were detected using electrospray ionization (ESI) sources, in both positive and negative ion modes, and the retention times (t) of each compound, the precursor and product ions, the cluster voltage (DP), and the collision energy (CE) were analyzed. In order to effectively distinguish isomers by high-resolution mass spectrometry and secondary mass spectrometry fragments, the individual solutions of all standard compounds (100 ng/mL in 70% (*v*/*v*) acetonitrile) were injected into the ESI source in the positive and negative ion modes to obtain more suitable declustered voltage (DP) and collision energy (CE) parameters. The most abundant fragment ions were chosen as MRM transition from MS/MS spectrum; after trial-and-error inspection, most constituents had a good response in the negative ion mode. The chromatogram, in multi-reaction monitoring (MRM) mode, is shown in Figure S1.

### 3.2. Verification of Analytical Methods

Table 3 lists the verification results obtained using this method. A standard calibration curve indicated that the determination coefficients for all analytes were good (r > 0.9990). The limit of detection (LOD) with the signal to noise ratio (S/N) was about 3. The limit of quantitation (LOQ) with the signal to noise ratio (S/N) was about 10. The limit of detection (LOD) and the limit of quantitation (LOQ) were 0.15–3.37 ng/mL and 0.49–13.77 ng/mL, respectively. Within days, 47 analytes were identified with relative standard deviation (RSD) values of 1.28–3.29% and 1.60–2.89%, respectively. The repetitive stability test value for all 47 components was less than 4, and the average recovery was between 94.89% and 105.42%, with an RSD% of less than 3.89% and the slope ratio of the matrix curve to the pure solution curve was 0.93–1.06, supporting the validity of the proposed method.

The quantitative determinations are shown in Table S1. In the current Chinese Pharmacopoeia, citric acid is considered to be the most characteristic component of MF. Based on the analysis of 18 batches of samples, the citric acid contents were found to be similar to those reported by the pharmacopeia standard, and 47 analytes, identified in 18 batches of samples, were analyzed using the verified analytical method. The contents of all 47 analytes are summarized in Table 4. The data from all samples indicate that MFd (178,111.72 µg/g) > MFf (163,284.84 µg/g). The contents of organic acids and polysaccharides (5-hydroxymethylfurfural) in MFd were higher than those in MFf. However, the contents of flavonoids (2597.40 µg/g) and amino acids (9947.03 µg/g) in the MFf were higher than those in the MFd (flavonoids: 2265.69 µg/g; amino acids: 6692.86 µg/g). By comparing these parameters, we observed that the contents and compositions of the MFd and MFf samples were quite different.

### 3.3. Principal Component Analysis (PCA)

**Figure 1:**
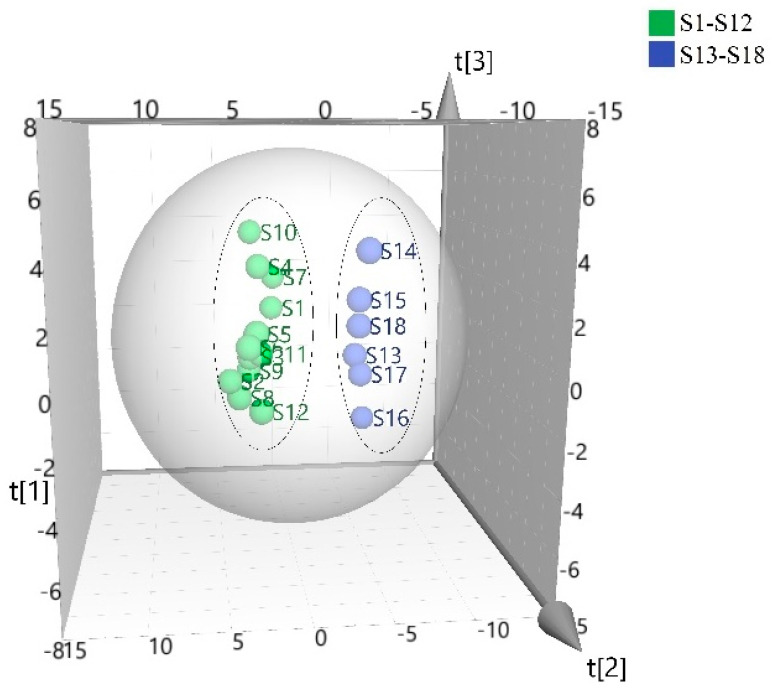
Scatter plot showing the score of PCA-processed data, acquired from the samples. Each of the blue circles represents a batch of MFf, whereas each green circle represents a batch of MFd.

PCA is a common data dimensionality reduction technique that can be applied to distinguish between samples and visualize clusters and outliers. According to the contents of the 47 components identified in the samples, PCA was used to distinguish between the samples of MF processed with different drying methods. After the raw data were standardized, SPSS, v. 23.0, was used to perform PCA. The eigenvalues and variance contributions of the principal components (PCs) can be found in Table 4. Among these, eight eigenvalues greater than 1 were identified, and the cumulative contribution rate was 84.63. The cube was simplified into a three-dimensional data set using three eigenvalues (PC1: 30.41%; PC2: 13.16%; and PC3; 9.59%). As shown in Figure 1, the samples can be divided into two categories (drying and fumigation), and the results show that the composition and contents of the dried samples differed greatly from those for fumigated samples.

### 3.4. Partial Least Squares Discriminant Analysis (PLS-DA) of the Samples

**Figure 2:**
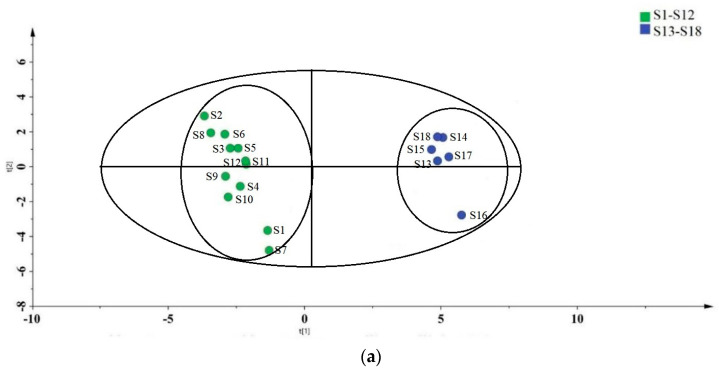

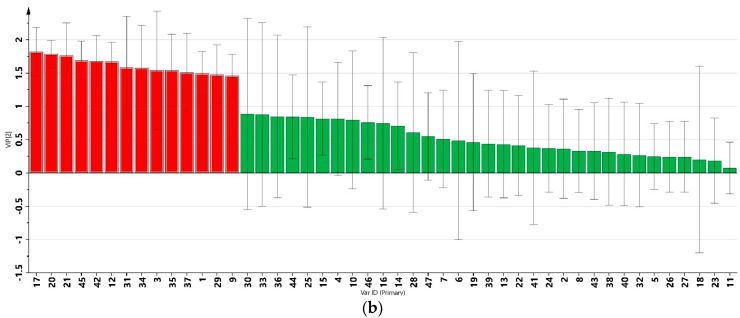
The MFf and MFd data were PLS-DA-treated and visualized on a scatter plot (**a**), followed by VIP (**b**) processing. Each blue circle indicates a batch of MFf, whereas each green circle indicates a batch of MFd. The VIP abscissa indicates the compounds (see Table 1 for the name of each compound). (Red represents VIP >1, green represents <1).

To identify potential chemical markers with significant impacts on sample identification, PLS-DA and variable importance in predictive trials (VIP) were used. The PLS-DA score and VIP values are shown in Figure 2a,b. Samples processed by drying and fumigation were also divided into two groups using these analyses, indicating significant differences in the chemical compositions of samples processed by MFd and MFf. The OPLS-DA results show that R2Y was 0.991, indicating that the model fitted well, and Q2 = 0.951 > 0.5 indicated that the model predicted well.

The VIP results describe the overall contribution of each variable to the model, with the threshold typically set to VIP greater than 1 to identify important variables. In this experiment, VIP values were obtained using the PLS-DA-processed data. Variables with VIP greater than 1 can be viewed as potential markers that contribute significantly to these sample classifications. For example, 5-hydroxymethylfurfural (17), *L*-aspartic acid (20), *L*-asparagine (21), uridine (45), thymidine (42), kaempferol (12), *L*-cystine (31), *L*-tryptophan (34), caffeic acid (3), *L*-proline (35), γ-aminobutyric acid (37), benzoic acid (1), *L*-phenylalanine (29), and rutin (9).

From Figure 2b, we can see that Compound 17 (5-hydroxymethylfurfural) has the highest VIP value. The Chinese Pharmacopoeia edition, only defines a limit for 5-hydroxymethylfurfural in glucose injections, which should not exceed 0.02% of the calculated mass fraction. The levels of 5-hydroxymethylfurfural identified in samples prepared using different processing methods in this study were much higher than this limit. In addition, the content of 5-hydroxymethylfurfural in MF was also much higher than the concentration used in cells, which demonstrated weak toxicity in previous studies and changed some indices in animal models [23,24]. Plums are used as both food and medicine in daily life, and the European Commission for Food Safety has established a maximum recommended intake of 1.6 mg of 5-hydroxymethylfurfural substances per person per day. However, 5-hydroxymethylfurfural can be widely found in various foods, resulting in the daily average intake being much higher than this recommended limit higher. Despite this intake, no strong evidence has supported 5-hydroxymethylfurfural as a health hazard. Therefore, the potential harm and limits of 5-hydroxymethylfurfural in Chinese medicinal materials, such as MF, requires further study.

## 4. Discussion

In this study, the UPLC-MS/MS technology achieved significant results by identifying 47 components in negative ion mode. We found that many compounds (such as apigenin, luteolin, and quercetin) exhibited higher sensitivity in negative ion mode, which is consistent with the characteristic of these compounds having acidic functional groups that easily undergo proton loss for ionization. Previous studies have also indicated that flavonoids have higher ionization efficiency in negative ion mode [25]. Furthermore, we effectively distinguished multiple pairs of isomers (such as L-leucine and L-isoleucine) using specific fragment information from high-resolution mass spectrometry and tandem mass spectrometry, further validating the reliability of the optimized ESI parameters (DP and CE values). The MRM chromatograms in Figure S1 demonstrate the good signal response and retention time separation of different compounds, indicating that the multiple-reaction monitoring mode used can effectively detect multiple target compounds simultaneously. A validated analytical method is fundamental for the efficient detection of chemical components in traditional Chinese medicine. The validation results of the methods in this study show that all analytes had a wide linear range (R^2^ > 0.9990), with limits of detection (LODs) and quantification (LOQs) reaching 0.15–3.37 ng/mL and 0.49–13.77 ng/mL, respectively, demonstrating sensitivity superior to many methods reported in the literature [26,27]. Additionally, the matrix effect test (0.93–1.06) and sample recovery rates (94.89–105.42%) indicated that the method is accurate and has a significant advantage of being unaffected by matrix interference. This result further confirms that the proposed method has high precision, stability, and reproducibility, making it widely applicable for the analysis of similar samples. The analysis of 18 batches of MEf (dried plum samples processed differently) revealed that the total organic acid content in dried samples (MFd) was significantly higher than that in fumigated samples (MFf), while the latter had higher levels of flavonoids and amino acids. This may be related to the impact of processing methods on the chemical composition of dried plums. For example, the drying process may lead to the partial degradation of flavonoids and amino acids, while fumigation heating may promote the production of certain metabolites [28]. Notably, the citric acid content in these results meets the standards set by the “Chinese Pharmacopoeia,” confirming its applicability as a quality marker and indicating that different processing methods significantly influence the composition distribution of medicinal materials. HMF is an important product in the processing of food and medicinal plants, and its content is only defined in the Chinese Pharmacopoeia for glucose injection (0.02%). However, the HMF levels in all samples of this study were far above this standard (Figure 2b). Although animal model studies suggest that HMF may have certain toxicity at higher concentrations, current conclusions regarding its safety are inconsistent, with the European Food Safety Authority recommending a daily intake of 1.6 mg. However, considering the widespread presence of HMF in dried plums and other edible medicinal materials, its long-term accumulation effects and actual impact on human health require further investigation. Additionally, this study proposes the potential of HMF as a marker for processing, but there is a lack of unified standard limits for HMF in traditional Chinese medicinal materials, highlighting the need to address this issue in the future standardization process of traditional Chinese medicine.

## 5. Conclusions

In this study, a UPLC-QTRAP-MS/MS method was established and validated as a rapid, convenient, and sensitive way for the simultaneous determination of 47 compounds in Mume Fructus, within 14 min. It was successfully applied to the quantification of the 18 samples of different drying methods of MF.

## Figures and Tables

**Table 1:** Information of tested MFf and MFd samples.

Samples	No.	Origin	Origin
MFf	S1	Sichuan	fumigation
	S2	Sichuan	
	S3	Sichuan	
	S4	Sichuan	
	S5	Sichuan	
	S6	Sichuan	
	S7	Fujian	
	S8	Fujian	
	S9	Fujian	
	S10	Fujian	
	S11	Fujian	
	S12	Fujian	
MFd	S13	Sichuan	drying
	S14	Sichuan	
	S15	Sichuan	
	S16	Sichuan	
	S17	Sichuan	
	S18	Sichuan	

MFf: Mume Fructus were fumigated; MFd: Mume Fructus were dried.

**Table 2:** Retention times and related mass spectrometry (MS) data for the target compounds.

No.	Name	Formula	(tR)	[M + H]+	[M − H]−	MRM	DP/V	CE/V
			(min)	m/z	m/z	(Precursor→Product)		
1	Benzoic acid	C_6_H_6_O_3_	10.43	-	120.92	120.5-76.9	−56	−16
2	Quercitrin	C_21_H_20_O_11_	9.70	-	447.81	447.2-299.8	−145	−35
3	Caffeic acid	C_9_H_8_O_4_	9.74	-	178.53	178.5-134.6	−68	−22
4	Fumaric acid	C_4_H_4_O_4_	7.22	-	115.01	114.5-70.9	−57	−13
5	Chlorogenic acid	C_16_H_18_O_9_	6.02	-	353.11	352.8-190.7	−79	−20
6	Protocatechuic acid	C_7_H_6_O_4_	6.29	-	152.95	152.6-108.7	−59	−22
7	Succinic acid	C_4_H_6_O_4_	4.21	-	116.82	116.3-72.8	−28	−17
8	Quininic acid	C_7_H_12_O_6_	5.47	-	190.52	190.5-85.0	−60	−27
9	Rutin	C_27_H_30_O_16_	8.87	-	610.55	609.0-300.6	−162	−48
10	Citric acid	C₆H₈O₇	6.82	-	190.32	190.3-74.8	−202	−25.9
11	Malic acid	C_4_H_6_O_5_	6.03	-	132.93	132.5-114.0	−69	−13
12	Kaempferol	C_15_H_10_O_6_	13.28	-	283.85	282.9-246.8	−120	−35
13	Ursolic acid	C_30_H_48_O_3_	10.89	457.37	-	457.3-411.2	116	40
14	Amygdalin	C_20_H_27_NO_11_	6.45	458.41	-	458.3-163.0	50	20
15	Cyanidin-3-O-glucoside chloride	C_21_H_21_O_11_	6.27	449.29	-	449.2-287.2	17	27
16	Apigenin	C_15_H_10_O_5_	12.76	274.59	-	274.0-87.9	79	32
17	5-hydroxymethylfurfural	C_6_H_6_O_3_	6.66	109.0	-	109.0-53.0	87	21
18	*L*-alanine	C_3_H_7_NO_2_	4.36	91.76	-	90.06-44.02	79	10
19	*L*-serine	C_3_H_7_NO_3_	6.18	106.11	-	106.05-59.99	67	8
20	*L*-aspartic acid	C_4_H_7_NO_4_	1.7	134.13	-	134.05-87.96	59	10
21	*L*-asparagine	C_4_H_8_N_2_O_3_	6.22	132.97	-	132.80-115.70	46	13
22	*L*-valine	C_5_H_11_NO_2_	3.38	118.74	-	118.09-72.06	67	8
23	*L*-glutamic acid	C_5_H_9_NO_4_	6.53	151.64	-	147.08-83.92	83	14
24	*L*-isoleucine	C_6_H_13_NO_2_	5.81	132.41	-	132.00-86.00	66	15
25	*L*-methionine	C_5_H_11_NO_2_S	3.41	151.13	-	150.06-104.03	91	10
26	*L*-arginine	C_6_H_14_N_4_O_2_	6.46	175.78	-	175.12-70.02	88	18
27	*L*-histidine	C_6_H_9_N_3_O_2_	6.52	156.74	-	156.08-110.03	95	16
28	*L*-threonine	C_4_H_9_NO_3_	5.70	120.56	-	120.30-76.80	54	11
29	*L*-phenylalanine	C_9_H_11_NO_2_	2.84	166.98	-	166.10-120.05	56	14
30	*L*-leucine	C_6_H_13_NO_2_	2.81	132.87	-	132.10-86.05	98	10
31	*L*-cystine	C_6_H_12_N_2_O_4_S_2_	7.02	241.03	-	240.80-151.90	71	18
32	*L*-hydroxyproline	C_5_H_9_NO_3_	2.89	134.45	-	133.80-71.80	52	25
33	*L*-tyrosine	C_9_H_11_NO_3_	3.56	182.95	-	182.16-136.08	46	17
34	*L*-tryptophan	C_11_H_12_N_2_O_2_	2.73	205.46	-	205.00-188.00	202	15
35	*L*-proline	C_5_H_9_NO_2_	4.13	116.43	-	116.07-70.02	68	10
36	L-lysine hydrochloride	C_6_H_15_CIN_2_O_2_	6.52	147.41	-	147.00-84.00	52	24
37	γ-aminobutyric acid	C_4_H_9_NO_2_	3.14	104.24	-	103.70-86.90	32	14
38	Guanine	C_5_H_5_N_5_O	4.56	152.11	-	151.80-135.0	62	24
39	Adenine	C_5_H_5_N_5_	1.93	136.94	-	136.06-119.00	51	24
40	Uracil	C_4_H_4_N_2_O_2_	2.44	113.73	-	113.04-70.00	111	21
41	Hypoxanthine	C_10_H_12_N_4_O_5_	3.96	269.78	-	269.00-137.05	46	15
42	Thymidine	C_10_H_14_N_2_O_5_	2.39	255.17	-	243.10-127.07	61	13
43	Guanosine	C_10_H_13_N_5_O_5_	5.23	284.78	-	284.30-152.00	62	15
44	Inosine	C_10_H_12_N_4_O_5_	3.66	269.51	-	269.00-137.05	46	15
45	Uridine	C_9_H_12_N_2_O_6_	4.72	255.13	-	244.90-113.00	103	13
46	Adenosine	C_10_H_13_N_5_O_4_	3.47	268.12	-	267.9-118.7	86	23
47	Cytidine	C_9_H_13_N_3_O_5_	4.74	244.51	-	244.09-94.65	61	10

MRM: multiple-reaction monitoring; DP: declustering potential; CE: collision energy.

**Table 3:** Regression equations, detection limits (LODs), quantity limits (LOQs), intrinsic and differential precision, stability, reproducibility recovery, and matrix effects of 47 components.

No.	Regression Equation	Linear Range (μg/mL)	R2	LoD (ng/mL)	LoQ (ng/mL)	Precision (RSD%)		Stability	Repeatability	Recovery		Matrix Effect
						Intraday	Interday	(RSD %, *n* = 6)	(RSD %, *n* = 6)	Mean	RSD%	
						(*n* = 6)	(*n* = 3)					
1	y = 2.97 × 10^5^x − 1.57 × 10^5^	0.12–12.24	0.9991	0.43	1.28	2.32	2.31	2.83	2.58	104.38	3.34	1.01
2	y = 2 × 10^6^x − 870.64	0.0011–1.11	0.9993	0.33	1.11	2.32	2.26	2.81	2.45	95.54	2.37	1.03
3	y = 7.50 × 10^5^x − 7 × 10^5^	1.21–121.75	0.9995	0.55	1.56	2.17	2.79	2.22	2.21	97.39	3.69	1.05
4	y = 5.32 × 10^5^x − 2 × 10^6^	2.64–264.12	0.9991	0.69	2.41	1.62	2.89	2.39	2.73	104.39	2.18	1.02
5	y = 7.02 × 10^5^x − 2 × 10^7^	0.065–130.23	0.9993	2.14	6.18	1.96	2.38	2.87	2.57	101.54	3.55	0.96
6	y = 8.03 × 10^5^x − 3 × 10^6^	0.13–130.47	0.9994	0.95	3.76	1.99	2.77	1.75	2.68	96.56	2.65	0.99
7	y = 1 × 10^6^x − 4 × 10^6^	0.12–240	0.9992	0.69	1.63	2.31	1.99	2.61	2.21	99.61	2.01	1.03
8	y = 6.94 × 10^5^x − 2 × 10^6^	1.34–268.79	0.9996	2.16	6.81	3.29	2.68	2.51	2.89	97.75	2.44	0.97
9	y = 5.55 × 10^4^x − 6.26 × 10^4^	0.071–141.84	0.9990	0.39	1.38	2.33	2.38	2.92	2.49	98.33	3.03	0.97
10	y = 2.66 × 10^5^x − 2 × 10^7^	0.123–12300	0.9991	0.47	1.12	2.79	1.78	1.44	2.11	98.58	2.29	0.97
11	y = 2.40 × 10^5^x − 2 × 10^6^	0.015–600.45	0.9995	0.41	13.77	2.35	2.41	2.27	2.23	102.31	2.59	1.01
12	y = 3.21 × 10^4^x − 1.23 × 10^4^	0.012–40.13	0.9996	0.91	2.87	1.28	2.31	1.86	2.68	95.02	2.18	0.95
13	y = 1.43 × 10^4^x − 6.26 × 10^4^	0.011–200.14	0.9994	0.37	1.23	1.56	2.01	2.46	1.74	97.35	2.56	0.98
14	y = 1814.8x − 1230.6	0.081–50.29	0.9995	0.57	1.75	2.05	2.89	2.34	2.74	100.57	2.67	1.02
15	y = 9.59 × 10^5^x − 7.12 × 10^5^	0.012–121.23	0.9996	0.89	3.16	2.38	2.12	2.79	2.95	96.52	2.82	0.99
16	y = 3.61 × 10^4^x − 4 × 10^6^	0.015–258.47	0.9991	0.60	1.98	2.06	2.68	1.82	2.75	97.41	2.91	0.94
17	y = 3.38 × 10^5^x − 1 × 10^6^	0.097–9.86	0.9990	0.78	2.43	1.51	2.59	2.12	2.88	102.23	2.61	0.96
18	y = 4.2 × 10^5^x − 6.22 × 10^4^	0.097–9.86	0.9995	0.83	3.87	2.21	2.36	1.76	2.89	98.03	3.89	0.98
19	y = 3242.7 x − 4.51y = 10^4^	0.12–126.31	0.9995	3.83	9.87	2.73	2.56	2.09	2.88	98.35	2.61	0.96
20	y = 2.50 × 10^4^x − 1.89 × 10^4^	0.136–280.17	0.9998	3.26	10.9	2.28	2.36	2.59	2.66	96.73	2.67	1.01
21	y = 4.31 × 10^4^x − 1.45 × 10^5^	0.15–156.85	0.9992	0.93	3.42	2.06	1.99	2.16	2.01	104.94	2.09	0.98
22	y = 2.76 × 10^5^x − 1 × 10^6^	0.59–108.27	0.9997	1.47	3.90	2.34	2.56	2.17	2.01	102.21	2.88	1.01
23	y = 3082.3x − 5105.7	0.16–80.17	0.9997	0.88	3.96	1.99	2.37	2.89	2.25	97.15	2.31	1.05
24	y = 5 × 10^6^x − 4 × 10^6^	0.287–28.07	0.9996	0.15	0.49	2.14	2.78	2.03	2.01	98.45	2.51	0.97
25	y = 4.36 × 10^5^x − 1.14 × 10^4^	0.012–10.68	0.9995	0.65	2.08	1.75	2.88	2.33	2.07	96.64	2.44	0.98
26	y = 2 × 10^6^x − 8.74 × 10^4^	0.012–20.43	0.9997	3.37	10.2	1.38	2.13	2.26	2.28	94.89	2.11	0.93
27	y = 8 × 10^4^x − 8.74 × 10^4^	0.17–95.14	0.9993	0.91	4.15	2.38	2.34	2.86	2.46	103.21	2.61	1.03
28	y = 6.89 × 10^5^x − 3.89 × 10^4^	0.21–60.74	0.9991	0.66	2.18	1.91	3.23	1.52	2.07	99.18	2.47	0.94
29	y = 2 × 10^6^x − 3.39 × 10^5^	0.14–14.63	0.9995	2.11	7.04	1.85	2.13	2.76	1.94	97.26	2.06	1.03
30	y = 3261.4x − 2766.7	0.17–34.96	0.9991	0.42	1.23	2.06	1.97	2.23	2.06	103.24	2.36	1.02
31	y = 2686.5x − 970.63	0.95–54.93	0.9996	0.34	1.34	2.65	2.08	2.57	2.08	105.42	2.80	1.02
32	y = 1 × 10^6^x − 2.78 × 10^5^	0.81–40.49	0.9995	0.36	1.46	2.51	2.04	2.44	2.04	96.50	2.78	1.04
33	y = 4 × 10^4^ − 3.12 × 10^5^	0.021–105.71	0.9991	0.19	0.64	2.81	1.98	1.77	2.43	99.53	2.14	1.01
34	y = 3.28 × 10^4^x − 5.23 × 10^4^	0.13–91.84	0.9990	0.23	0.91	2.26	2.78	1.13	2.19	98.36	2.07	1.02
35	y = 8.21 × 10^5^x − 6.84 × 10^4^	0.214–30.69	0.9993	1.72	5.43	2.27	2.51	2.20	2.51	98.36	2.20	1.06
36	y = 1 × 10^7^x + 4 × 10^6^	0.41–160.34	0.9994	1.74	5.73	2.80	2.60	2.72	2.60	105.43	2.37	1.03
37	y = 4.68 × 10^4^x − 9.48 × 10^4^	0.161–66.43	0.9997	0.43	1.35	2.64	2.14	2.56	2.14	102.56	2.84	0.97
38	y = 2 × 10^7^x − 4 × 10^6^	0.056–25.14	0.9991	0.55	1.63	2.75	2.50	2.67	2.50	97.53	1.73	1.00
39	y = 8 × 10^6^x + 1.23 × 10^4^	0.089–3.74	0.9994	0.47	1.49	2.27	1.79	2.20	1.79	100.61	2.58	1.04
40	y = 3.46 × 10^5^x − 3.54 × 10^4^	0.056–1.89	0.9995	0.76	2.34	2.97	2.41	2.88	2.41	98.73	2.49	0.98
41	y = 1 × 10^7^x − 1 × 10^6^	0.13–8.78	0.9993	0.88	2.75	2.55	2.14	2.48	2.14	99.31	2.89	0.98
42	y = 3 × 10^6^x − 5.97 × 10^5^	0.23–46.21	0.9992	2.61	8.47	2.16	1.60	2.10	1.60	99.57	1.43	0.98
43	y = 1.09 × 10^5^x − 3.63 × 10^4^	0.107–20.89	0.9996	0.28	0.96	2.29	2.17	2.22	2.17	103.33	2.25	1.02
44	y = 2 × 10^7^x − 3.38 × 10^5^	0.018–37.42	0.9995	1.12	3.96	2.75	2.08	2.67	2.08	95.97	1.84	0.96
45	y = 7.59 × 10^4^x − 189.53	0.031–6.84	0.9994	2.31	6.72	1.78	1.81	1.73	1.81	98.32	2.44	0.99
46	y = 3 × 10^7^x + 3.20 × 10^5^	0.062–1.954	0.9991	0.22	0.71	2.81	2.60	2.73	2.60	101.58	2.32	1.03
47	y = 9 × 10^6^x − 4.62 × 10^5^	0.074–14.19	0.9993	1.21	3.43	2.97	1.91	2.94	1.91	97.49	2.76	1.00

**Table 4:** Contribution rate of principal components.

PCs	Eigenvalues	Variance %	Accumulate %
1	12.772	27.175	27.175
2	5.699	12.126	39.301
3	4.037	8.588	47.889
4	3.992	8.494	56.383
5	3.766	8.014	64.397
6	2.846	6.056	70.453
7	2.606	5.545	75.998
8	2.332	4.962	80.96

## Data Availability

The data used to support the findings of this study are available from the corresponding author upon request.

## Supplementary Materials

The following supporting information can be downloaded at: https://www.mdpi.com/article/10.3390/metabo15050312/s1, Figure S1: MRM chromatograms of forty-seven components.; Table S1: Determination of each sample (n = 3).

## References

[1] Chinese Pharmacopoeia Commission (2015). Chinese Pharmacopoeia Commission Pharmacopoeia of the People’s Republic of China. Part I.

[2] Ou J.M., Wang R., Li X.L., Huang L.Q., Yuan Q.J., Fang C.W., Wu D.L. (2020). Comparative Analysis of Free Amino Acids and Nucleosides in Different Varieties of Mume Fructus Based on Simultaneous Determination and Multivariate Statistical Analyses. J. Int. Anal. Chem..

[3] Zhang H.Y., Li Q., Fu X.L. (2017). Research progress on chemical constituents and pharmacological effects of Mume Fructus. Shanghai J. Trad. Chin. Med..

[4] Turturica M., Stanciuc N., Bahrim G., Rapeanu G. (2016). Effect of thermal treatment on phenolic compounds from plum (*Prunus domestica*) extract—A kinetic study. J. Food Eng..

[5] Khan A., Pan J.H., Cho S., Lee S., Kim Y.J., Park Y.H. (2017). Investigation of the hepatoprotective effect of *Prunus mume* Sieb.et Zucc extract in a mouse model of alcoholic liver injury through high-resolution metabolomics. J. Med. Food.

[6] Nirmal S.A., Pal S.C., Mandal S.C., Patil A.N. (2012). Analgesic and antiinflammatory activity of β-sitosterol isolated from nyctanthes arbortristis leaves. J. Inflammopharmacol..

[7] Takashi H., Hitoshi T., Atsushi N., Eri K., Sanae U., Naomi S., Tomohiro K., Ken S., Satoru K. (2013). Advanced hepatocellular carcinoma responds to MK615, a compound extract from the Japanese apricot “*Prunus mume*”. World J. Hepatol..

[8] Sriwilaijaroen N., Kadowaki A., Onishi Y., Gato N., Ujike M., Odagiri T., Tashiro M., Suzuki Y. (2011). Mumefural and related HM-F derivatives from Japanese apricot fruit juice concentrate show multiple inhibitory effects on pandemic influenza A (H1) A (H1N1) virus. Food Chem..

[9] Li J.L., Tang Q., Chen G. (2012). Study on the bacteriostatic activity, antiinflammation, analgesic and antipyretic effects of extract from Lonicera bud. Sci. Technol. Food. Ind..

[10] Liu Y.X., Bai J.X., Li T., Fu X.-Q., Guo H., Zhu P.-L., Chan Y.-C., Chou J.-Y., Yin C.-L., Li J.-K. (2019). A TCM formula comprising *Sophorae* Flos and *Lonicerae Japonicae* Flos alters compositions of immune cells and molecules of the STAT3pathway in melanoma microenvironment. Pharmacol. Res..

[11] He F., Xu B.L., Chen C., Jia H.J., Wu I.J.X., Wu X.C. (2016). Methylophiopogonanone A suppresses ischemia/reperfusion-induced myocardial apoptosis in mice via activating PI3K/Akt/eNOS signaling pathway. J. Acta. Pharmacol. Sin..

[12] Wang Y., Liu F., Liang Z., Peng L., Wang B.Q., Yu J., Su Y.Y. (2017). Homoisoflavonoids and the antioxidant activity of *Ophiopogon japonicus* root. J. Iran. J. Pharm. Res..

[13] Zhao R.H., Duan J.A., Gao Z.J., Zeng Y., Qian D.W., Su S.L., Zhou H.Y. (2013). Analysis and evaluation of traditional and modern drying technologies and methods of primary processing of traditional Chinese medicinal materials. J. Mod. Chin. Med..

[14] Duan J.A., Su S.L., Lv J.L., Yan H., Ding A.W. (2009). Traditional experiences and modern cognition on primary processing of traditional Chinese medicinal materials. J. Chin. Mater. Med..

[15] Ding C., Li B.S. (2012). Variation in Color during Smoking of Green Plum. J. Mod. Food Sci. Technol..

[16] Cao F., Kang Z.P., Xie X.M., Wu B.L., Zhang L. (2017). Determination and Analysis of Water Soluble Organic Acid in 5Rosaceae Fruit Medicines with Flavor Acid. J. Beijing Univ. TCM.

[17] Chen Z.G., En B.T., Zhang Z.Q. (2006). RP—HPLC Simultaneous Determination of 8Organic Acids in Mume Fructus. J. Chin. Mater. Med..

[18] An M., Li X., Zhao Y., Li G., Lu C.J., Wu Q.G. (2017). HPLC-PDA Simultaneous Determination of Three Characteristic Components in Mume Fructus. Chin. J. Exp. Tradit. Med. Form..

[19] Wu Q.S., Wang C.M., Lu J.J., Lin L.G., Chen P., Zhang Q.W. (2017). Simultaneous determination of six saponins in *Panax Japonici* Rhizoma using quantitative analysis of multi-components with single-marker method. J. Curr. Pharm. Anal..

[20] Wang X.P., Wang F., Bai J.J., Wang J., Ye Z.R. (2017). HPLC Simultaneous Determination of Four Saponins in Fujizhu Weikang Granules. J. Chin. J. New. Drugs Clin. Rein..

[21] Tan M.X., Chen J.L., Zhou L.S., Liu X.H., Tang R.M., Ma J.M. (2019). Effects of different drying methods on multiple bioactive constituents of Ophiopogonis Radix. J. Nat. Prod. Res. Dev..

[22] Wang R., Cheng H., Yang Y.T., Ou J.M., Song Q.Q., Zhou H.Y. (2022). Ultra-performance liquid chromatography-quadrupole-time of flight tandem-mass spectrometry and liquid chromatograph tandem mass spectrometer combined with chemometric analysis an approach for the quality evaluation of Mume Fructus. J. Sep. Sci..

[23] Fan W.G., Verrier F., Queneau Y., Popowycz F. (2019). 5-Hydroxymethylfurfural (HMF) in Organic Synthesis: A Review of its Recent Applications Towards Chemicals. J. Curr. Fine Org. Synth..

[24] Li M.M., Wu L.Y., Zhao T., Wu K., Xiong L., Zhu L.L., Fan M. (2011). The protective role of 5-hydroxymethyl-2-furfural (5-HMF) must exact hyperbaric hypoxia. J. Cell Stress. Chaperones.

[25] Lu H.Z., Ding Y., Wang J.Y., Chen C., Yao X.R., Yuan X.M., Bu F., Bao H., Dong Y.W., Zhou Q. (2024). Early administration of Wumei Wan inhibit myeloid-derived suppressor cells via PI3K/Akt pathway and amino acids metabolism to prevent Colitis-associated Colorectal Cancer. J. Ethnopharmacol..

[26] Sun J., LaMei X., Ansi A.W., Fan M., Li Y., Qian H., Fan L., Wang L. (2025). Metabolic profiling and amino acid evolution in fermented oats: Insights from UPLC-MS/MS and PLS-DA analysis. Food Biosci..

[27] Ren M., Wang Y., Yuan Y., Du H., Liang Q., Qin F., Xiong Z. (2025). Integration of UHPLC-MS and mass spectrometry imaging techniques revealed the protective mechanism of Gushudan in postmenopausal osteoporosis rats via branched-chain amino acid metabolism based on the ‘kidney-bone’ axis. J. Chromatogr. B.

[28] Shinde B., Patil D., Kadam N., Gautam M., Banerjee K., Gairola S., Doshi P. (2025). Ultra-high performance liquid chromatography with tandem mass spectrometry (UPLC-MS/MS) for simultaneous estimation of residual glyphosate and its metabolite (amino methyl phosphonic acid—AMPA) in various vaccines. Biol. J. Int. Assoc. Biol. Stand..

