# Supplementary material for: Determination of Multiple Active Components in Mume Fructus by UPLC-MS/MS
Source: Metabolites. 2025 May 6;15(5):312. doi: 10.3390/metabo15050312 (PMC12113456; doi:10.3390/metabo15050312)
Supplement: Supplementary file 1 [file metabolites-15-00312-s001.zip › metabolites-3557796-supplementary.pdf]

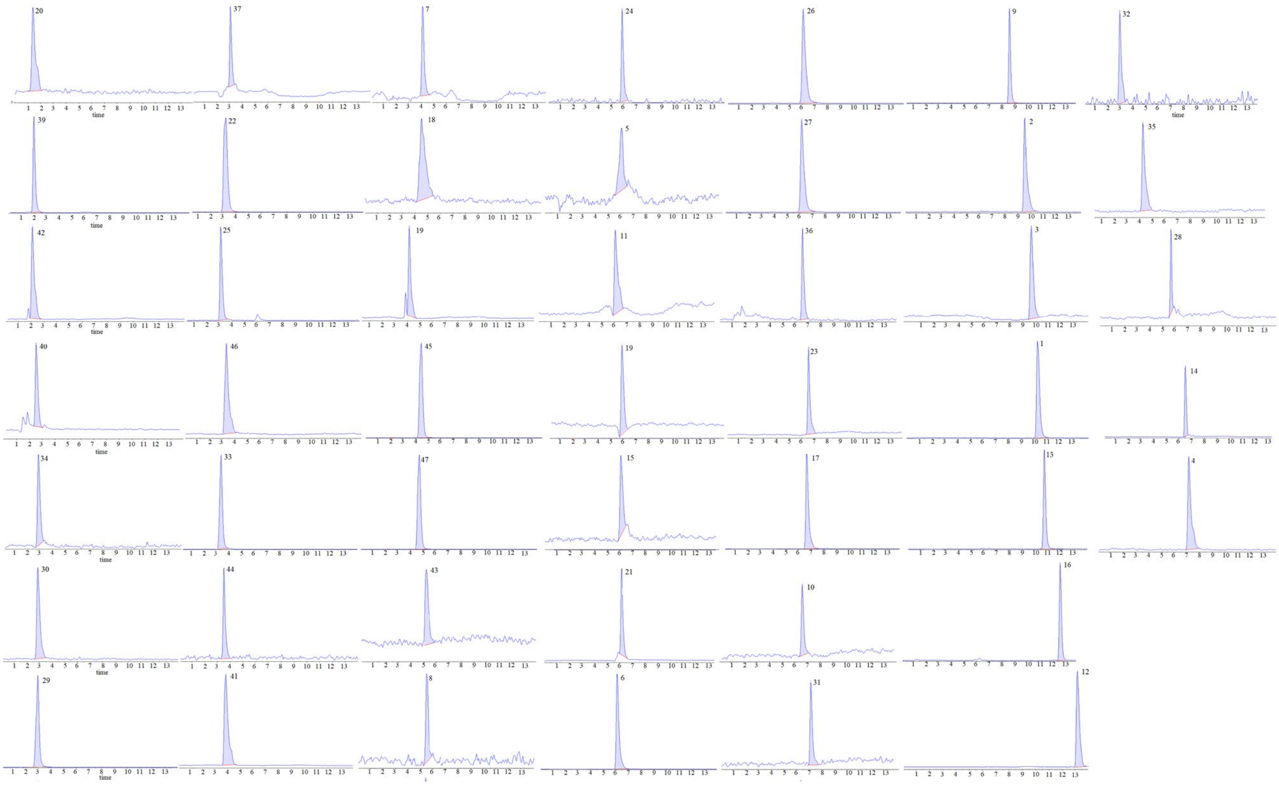

Figure S1. MRM chromatograms of forty-seven components.

Table S1. Determination of each sample(n = 3).

NO.	1	2	3	4	5	6	7	8	9	10
S1	78.73±2.01	0.05±0.09	1020.69±97.14	2455.47±81.94	445.96±40.72	33.47±1.53	806.22±102.19	2417.45±201.34	32.96±3.41	132801.33±1829.91
S2	77.32±1.89	0.06±0.07	1138.17±95.19	2585.65±117.02	440.04±45.12	30.06±0.99	1507.53±89.23	1703.43±49.12	44.32±2.13	108501.10±1232.54
S3	95.53±1.98	0.68±0.19	1306.09±101.23	2652.43±105.11	356.77±37.19	30.29±0.58	1676.13±102.65	1804.79±141.81	51.76±1.53	154506.34±2187.53
S4	75.92±2.11	0.07±0.11	1187.85±104.12	2355.88±141.32	431.16±16.94	28.36±1.22	1554.31±121.12	1588.73±112.31	46.14±1.42	144911.28±2013.98
S5	82.12±2.10	0.06±0.09	1304.46±88.12	1984.59±132.10	398.47±29.18	28.97±1.29	1528.72±189.25	1447.33±35.51	49.00±0.99	149966.63±2121.01
S6	81.74±2.23	0.07±0.0	1350.78±103.	1839.89±173.	399.61±49.	31.64±3.	1776.08±165.	1415.06±211.	49.45±1.	153397.67±2167.2

		2	98	45	43	01	97	37	32	2
S7	88.55±2.33	0.052±0.05	1027.86±111.21	2444.42±273.18	460.90±29.51	32.49±1.11	1545.28±142.93	1907.41±159.12	41.55±2.31	112481.70±3104.43
S8	92.24±1.96	0.07±0.09	1142.1478.30	2571.68±319.23	445.39±52.12	31.55±0.46	1531.52±71.05	1813.46±201.41	54.33±1.89	106179.12±4012.96
S9	109.35±2.46	0.67±0.18	1321.10±72.16	2663.41±201.58	349.76±20.12	33.28±0.71	1790.32±64.18	1924.88±102.41	55.23±1.32	144418.33±2971.91
S10	91.92±2.89	0.08±0.20	1171.85±78.22	2655.89±207.47	391.19±16.48	28.63±1.92	1651.10±118.29	1671.77±98.12	49.40±2.84	124643.22±3245.12
S11	96.32±1.92	0.07±0.07	1328.48±85.99	2184.53±89.58	368.41±42.69	29.71±1.67	1563.70±114.42	1647.45±75.92	51.81±1.93	150041.30±1692.65
S12	95.64±0.91	0.06±0.02	1345.74±72.15	1997.84±81.98	405.70±36.06	30.67±1.16	1876.79±127.19	1635.61±94.35	52.56±2.01	161217.61±2941.68
S13	61.32±1.21	0.21±0.09	1523.71±33.89	1938.34±147.98	381.73±36.81	29.41±0.55	1737.91±123.21	1570.79±120.54	34.02±3.29	159162.30±2491.92
S14	60.72±1.90	0.07±0.08	1627.90±94.54	1880.02±131.29	467.34±23.14	26.80±0.92	1501.24±100.23	1551.40±114.39	33.21±3.01	171467.41±2091.76
S15	72.14±2.78	0.06±0.02	1734.34±90.12	1872.87±109.29	465.23±39.23	27.12±2.01	1726.95±155.01	1585.62±113.82	37.08±3.29	163292.14±2913.91
S16	66.09±3.01	0.07±0.03	1503.01±94.54	2299.08±176.12	391.57±38.96	36.47±1.32	1698.30±191.13	1808.4±201.31	33.35±4.01	149753.31±1934.25
S17	65.83±1.23	0.06±0.04	1554.49±104.22	2325.70±199.21	403.51±63.12	38.04±2.41	1724.69±109.23	1842.59±149.24	34.94±2.94	139591.68±2259.47
S18	61.35±2.31	0.06±0.02	1472.42±99.13	2257.88±156.29	398.76±49.34	37.16±1.99	1648.64±111.31	1809.86±198.11	34.23±4.12	140152.12±1923.56

NO.	11	12	13	14	15	16	17	18	19	20
S1	2586.03±134.29	527.57±12.65	2742.24±102.33	736.17±50.29	33.05±1.01	1875.99±101.23	826.76±22.19	9.73±0.11	682.11±70.12	2577.01±231.41

S2	2533.81±218.29	590.96±33.34	3080.72±133.49	579.89±62.52	35.48±0.67	2109.74±41.29	837.94±35.12	9.501±0.07	852.78±64.21	2262.97±213.98
S3	2867.88±217.21	502.80±34.82	3001.83±139.32	611.83±98.31	34.07±0.26	2065.29±79.21	880.98±31.45	9.49±0.02	712.49±59.53	2410.76±312.45
S4	2464.21±116.29	506.93±40.59	3032.53±201.78	699.75±68.62	34.07±0.16	2036.43±123.65	924.28±29.92	9.47±0.12	680.59±67.59	2521.91±289.13
S5	2852.02±141.54	530.10±23.01	2987.67±172.19	451.50±49.59	34.61±0.31	1885.66±123.52	844.97±13.92	8.19±0.07	615.54±37.19	2225.58±199.54
S6	2975.33±162,45	549.95±17.58	3194.71±184.45	691.18±57.56	34.50±0.23	2159.60±93.35	888.33±29.18	9.57±0.02	615.55±41.12	2230.08±241.25
S7	2402.27±178.59	531.70±9.87	2689.36±194.67	729.67±50.31	32.50±0.97	1869.93±119.49	815.65±22.88	9.30±0.64	695.09±54.25	2317.14±321.54
S8	2413.01±164.28	581.96±13.04	3045.80±129.42	568.88±64.92	34.80±0.43	2101.39±176.56	841.37±30.29	9.08±0.21	615.82±91.23	2214.69±267.19
S9	2537.85±102.35	510.88±41.29	3089.26±219.52	622.80±65.29	31.67±0.58	2013.90±58.19	889.75±22.19	9.90±0.31	701.92±81.23	2211.68±112.52
S10	2144.14±189.41	506.93±45.19	3032.53±189.23	691.79±56.94	34.68±1.11	2025.94±42.91	889.84±41.21	9.70±0.35	691.86±67.94	2401.11±301.41
S11	2552.15±267.49	530.10±59.01	3081.66±181.20	442.04±67.25	32.10±1.22	1897.57±102.39	864.68±61.56	8.89±0.27	601.43±75.59	2105.76±102.46
S12	2815.11±274.25	541.97±39.12	3014.04±219.21	680.78±74.71	33.02±0.97	2134.00±73.13	892.33±52.41	9.69±0.19	605.50±76.26	2191.77±197.92
S13	2740.95±231.97	420.62±59.21	2811.08±234.12	493.52±59.23	33.03±1.22	1919.33±218.53	1371.17±71.25	10.44±0.06	1060.27±102,45	1023.82±201.23
S14	2538.87±99.12	449.84±42.19	3143.74±267.56	542.03±38.86	32.92±0.92	1373.68±201.56	1483.91±86.41	11.15±0.49	693.069±82.49	1070.49±223.41
S15	2746.75±201.24	427.73±34.23	2965.72±99.24	627.20±63.39	31.49±1.29	2210.12±152.98	1532.85±91.24	9.64±0.06	681.81±79.23	1065.01±273.49
S1	2570.40±219.	385.13±43.	2744.32±123.	615.82±75.	32.53±1.	1337.29±189.	1434.82±87.9	4.93±0.0	644.79±82.13	664.78±189.4

6	92	54	42	12	09	68	3	3		5
S17	2621.11±124.36	407.66±65.21	2937.44±89.13	558.29±81.25	33.79±0.45	2159.36±212.42	1420.13±79.11	10.75±0.07	646.33±74.22	693.41±78.26
S18	2449.14±196.23	389.35±17.29	3159.47±246.21	495.26±82.34	32.27±0.53	1906.70±204.51	1464.65±76.15	10.62±0.05	626.54±69.95	1005.28±241.12

NO.	21	22	23	24	25	26	27	28	29	30	31
S1	1935.08±143.25	743.49±82.45	426.45±35.23	71.26±3.32	0.82±0.013	5.21±0.23	130.31±8.12	22.75±1.24	919.19±101.46	32.61±1.32	482.54±23.98
S2	1942.9±104.37	962.49±79.39	269.77±41.51	74.46±5.23	0.81±0.034	5.19±0.67	129.83±16.23	20.26±2.15	1092.80±128.23	30.35±1.43	419.19±29.12
S3	1868.55±201.47	905.62±81.65	477.72±29.48	74.28±4.32	0.82±0.021	4.72±0.18	118.12±14.85	20.19±2.91	1040.04±129.45	31.22±2.31	490.43±42.16
S4	1879.67±98.12	740.86±87.53	447.42±24.63	72.98±3.56	0.79±0.067	4.63±0.27	115.84±19.21	24.00±1.35	1049.83±112.42	28.74±2.89	478.20±44.69
S5	1616.55±142.89	923.89±56.69	362.36±35.74	73.20±5.35	0.81±0.042	5.10±0.19	127.52±13.66	16.22±3.13	955.86±97.24	28.93±1.56	418.01±40.25
S6	1887.54±174.45	919.29±101.81	381.80±43.67	77.55±9.25	0.74±0.035	5.19±0.29	129.85±8.94	22.97±4.19	1082.19±88.27	30.50±1.44	442.64±38.44
S7	1901.82±201.32	703.87±86.24	426.45±21.85	73.29±10.12	0.81±0.039	4.13±0.53	103.17±7.71	21.52±3.57	910.88±91.56	31.07±1.32	472.38±35.85
S8	1921.08±129.43	951.85±76.49	269.77±43.95	73.59±8.92	0.83±0.019	5.93±0.69	148.30±9.64	21.57±2.52	1113.00±104.29	29.50±2.49	469.88±27.41
S9	1902.47±89.23	915.18±103.96	477.72±31.44	71.78±5.91	0.84±0.083	4.25±0.71	106.17±10.42	21.86±1.59	1141.37±111.23	30.92±2.34	470.33±27.97
S10	1809.74±229.44	713.55±98.39	447.42±34.42	74.75±4.23	0.78±0.039	4.34±0.85	108.41±11.34	22.96±1.95	1019.29±97.43	28.38±1.28	467.02±32.51
S11	1601.50±67.23	913.86±57.45	362.36±19.21	72.98±5.46	0.71±0.051	5.01±0.43	125.21±13.61	28.18±1.62	995.60±63.32	28.35±2.45	421.10±27.92

S12	1717.45±69.04	819.95±65.93	381.80±13.25	73.47±7.27	0.71±0.065	5.94±0.56	148.53±12.43	21.71±3.91	1032.87±92.19	29.97±3.11	460.40±16.98
S13	925.53±19.29	772.02±49.23	383.36±24.59	70.71±1.43	0.72±0.032	4.56±0.86	114.11±18.95	20.70±3.15	807.02±95.23	29.56±3.15	527.14±19.24
S14	342.53±42.94	880.05±63.35	480.10±21.24	72.31±1.94	0.72±0.057	5.76±0.91	143.90±17.48	21.41±4.94	791.48±82.51	33.02±2.43	582.82±31.18
S15	483.314±41.49	848.34±73.52	438.71±29.57	72.93±4.29	0.76±0.032	5.30±0.89	132.54±13.54	25.39±2.18	852.19±91.32	28.75±2.19	581.79±32.43
S16	637.81±85.23	789.92±69.47	333.58±32.12	73.02±7.32	2.07±0.022	4.60±0.68	115.01±19.36	29.32±2.24	852.23±65.46	31.64±2.92	537.25±25.16
S17	924.50±149.56	855.73±91.24	309.34±29.55	74.45±8.47	2.62±0.035	5.24±0.76	130.91±13.15	23.30±2.78	872.69±94.64	33.73±2.34	554.57±28.54
S18	439.91±52.76	848.32±86.78	396.16±40.26	74.96±6.19	0.78±0.067	5.06±0.47	126.38±16.54	23.89±3.21	925.8±96.11	35.26±2.52	656.17±30.43

NO.	32	33	34	35	36	37	38	39	40	41	42	43
S1	662.92±47.53	248.54±16.24	6.78±0.11	12.11±1.28	515.21±21.53	55.47±3.54	3.54±0.13	5.53±0.82	12.44±0.31	5.40±0.23	8.32±0.54	31.20±1.57
S2	548.64±51.24	255.59±21.47	6.14±0.12	15.03±1.23	446.54±23.98	54.70±2.64	3.76±0.53	5.14±0.34	10.13±0.42	6.74±0.43	8.82±0.36	34.45±1.21
S3	566.97±49.43	260.51±18.32	6.78±0.15	13.92±2.01	543.91±16.23	55.02±2.93	3.97±0.21	5.62±0.12	16.36±0.53	5.08±0.22	8.69±0.34	33.50±1.02
S4	734.70±43.29	247.80±9.13	6.35±0.21	13.02±2.41	511.02±28.12	56.02±4.21	3.59±0.34	6.74±0.91	18.95±0.64	6.62±0.41	8.44±0.36	31.95±1.34
S5	684.85±31.55	256.18±34.23	5.98±0.05	13.16±1.12	446.37±31.53	54.43±3.12	3.49±0.55	6.90±0.45	19.69±0.23	6.78±0.25	8.69±0.69	34.32±2.09
S6	513.03±29.34	252.30±19.55	6.48±0.09	14.21±0.94	514.12±33.15	56.35±4.09	3.64±0.22	7.26±0.62	19.33±0.15	6.64±0.56	8.91±0.81	33.16±2.14
S	612.2	241.3	6.79	11.1	497.0	49.6	3.42	6.28	18.4	6.02	8.24	30.0

7	1±23.	5±15.	±0.0	7±1.	7±30.	8±1.	±0.1	±0.5	2±0.	±0.2	±0.3	1±1.
	12	21	8	43	19	25	6	1	32	9	7	65
S	558.4	250.9	6.35	14.2	456.3	51.9	3.61	5.55	16.2	5.40	8.47	31.4
8	2±35.	5±17.	±0.1	8±1.	7±31.	8±1.	±0.5	±0.3	9±0.	±0.3	±0.6	7±2.
	23	55	5	96	53	98	3	5	56	9	4	32
S	596.7	242.0	6.82	14.2	533.0	52.2	3.74	5.22	16.6	5.83	8.91	32.0
9	4±36.	5±14.	±0.3	4±0.	7±34.	0±1.	±0.1	±0.7	4±0.	±0.5	±0.2	0±1.
	51	81	2	54	17	23	9	6	23	3	3	22
S	714.9	251.0	6.50	13.2	571.2	51.2	3.91	5.42	17.3	6.52	9.14	31.4
1	9±37.	4±17.	±0.1	6±2.	2±25.	3±2.	±0.6	±0.8	3±0.	±0.6	±0.7	6±1.
0	25	23	5	77	63	42	8	3	56	4	3	89
S	694.4	241.7	5.81	12.5	466.6	51.3	3.93	6.03	12.9	6.72	9.91	35.2
1	6±29.	6±19.	±0.2	6±2.	8±39.	3±2.	±0.5	±0.9	4±0.	±0.3	±0.7	2±1.
1	51	42	3	79	15	64	3	6	39	1	9	65
S	503.3	242.9	6.85	14.0	504.2	52.5	2.44	5.57	14.3	6.54	9.96	34.5
1	4±25.	6±23.	±0.4	6±2.	0±41.	5±3.	±0.7	±0.5	0±0.	±0.0	±0.2	5±1.
2	25	59	5	15	69	51	5	7	42	7	4	46
S	612.3	223.2	8.05	10.0	537.9	46.7	3.34	5.09	14.5	5.16	10.9	34.2
1	1±27.	6±25.	±0.3	7±1.	7±39.	1±3.	±0.6	±0.5	8±0.	±0.0	0±0.	4±2.
3	62	23	2	68	18	69	3	5	51	3	29	45
S	732.0	251.6	7.30	10.8	634.8	50.6	4.11	6.39	17.4	6.46	10.9	31.5
1	0±50.	4±10.	±0.3	9±1.	7±37.	0±3.	±0.7	±0.9	8±0.	±0.1	5±0.	8±2.
4	21	58	4	34	16	42	4	1	53	2	13	19
S	686.3	245.8	7.45	10.5	606.6	47.2	3.71	5.77	16.9	6.22	10.9	31.8
1	5±21.	6±32.	±0.6	5±2.	5±54.	1±2.	±0.4	±0.9	7±0.	±0.2	7±0.	9±1.
5	78	15	9	41	22	47	5	4	63	1	28	39
S	481.6	223.1	7.90	9.84	405.8	42.4	3.42	5.05	17.6	5.57	10.9	31.5
1	5±46.	5±29.	±0.2	±3.6	9±22.	2±3.	±0.8	±0.9	2±0.	±0.0	2±0.	3±2.
6	26	41	1	2	41	16	6	2	56	9	12	14
S	634.6	225.9	7.31	10.6	622.4	43.8	3.70	5.38	18.3	6.06	11.5	29.3
1	4±39.	2±24.	±0.6	7±0.	0±39.	5±2.	±0.4	±0.8	1±0.	±0.0	0±0.	3±2.
7	54	65	5	58	41	23	5	8	12	8	14	31
S	692.4	259.1	7.91	11.2	554.6	47.6	3.87	6.05	16.0	6.22	11.6	34.8
1	7±47.	3±28.	±0.5	4±0.	0±37.	7±5.	±0.5	±0.7	5±0.	±0.2	3±0.	3±1.
8	17	31	7	99	21	32	8	5	43	5	19	46

NO.	44	45	46	47
S1	0.49±0.01	14.03±1.01	1.66±0.08	1.87±0.12
S2	0.50±0.08	15.61±1.73	1.83±0.04	2.04±0.11
S3	0.51±0.03	15.48±1.31	1.75±0.05	1.93±0.18
S4	0.49±0.04	14.99±0.92	1.59±0.07	1.92±0.09
S5	0.50±0.02	14.34±0.89	1.68±0.09	1.91±0.08
S6	0.50±0.04	16.09±0.78	1.68±0.12	1.93±0.18
S7	0.41±0.09	15.33±1.43	1.62±0.08	1.66±0.08

S8	0.52±0.03	14.12±1.09	1.84±0.09	2.36±0.11
S9	0.52±0.07	14.18±1.03	2.78±0.07	1.27±0.13
S10	0.44±0.09	15.89±1.35	1.55±0.11	1.15±0.12
S11	0.48±0.05	15.36±1.29	2.51±0.12	1.12±0.07
S12	0.49±0.02	14.92±0.81	2.78±0.07	1.80±0.09
S13	0.46±0.06	11.97±0.67	1.53±0.06	1.89±0.11
S14	0.46±0.04	11.95±1.22	1.62±0.05	1.95±0.04
S15	0.46±0.05	12.01±0.53	1.57±0.11	1.90±0.06
S16	0.46±0.06	11.13±0.99	1.56±0.12	1.87±0.09
S17	0.46±0.07	12.29±1.14	1.60±0.11	2.01±0.07
S18	0.46±0.08	12.40±1.31	1.63±0.11	1.99±0.08
